# Anterior Cruciate Ligament Injury: Conservative Versus Surgical Treatment

**DOI:** 10.7759/cureus.20206

**Published:** 2021-12-06

**Authors:** Kevin Rodriguez, Mridul Soni, Pranay K Joshi, Saawan C Patel, Devarashetty Shreya, Diana I Zamora, Gautami S Patel, Idan Grossmann, Ibrahim Sange

**Affiliations:** 1 Research, Universidad Americana (UAM) Facultad de Medicina, Managua, NIC; 2 Research, Shri Lal Bahadur Shastri Government Medical College, Mandi, IND; 3 Research, Department of Medicine, Byramjee Jeejeebhoy (BJ) Medical College, Ahmedabad, IND; 4 Medicine, Pramukhswami Medical College, Karamsad, IND; 5 Research, Gandhi Medical College and Hospital, Secunderabad, Secunderabad, IND; 6 General Medicine, Universidad de Ciencias Médicas Andrés Vesalio Guzman, San José, CRI; 7 Internal Medicine, Pramukhswami Medical College, Karamsad, IND; 8 Research, Medical University of Silesia in Katowice Faculty of Medical Sciences Katowice, Katowice, POL; 9 Research, Karamshi Jethabhai (KJ) Somaiya Medical College, Mumbai, IND

**Keywords:** acl instability, anterior cruciate ligament (acl) injuries, acl tear, conservative and surgical treatment, orthopedic sports medicine, ortho surgery, acl injury, anterior cruciate ligament (acl) reconstruction

## Abstract

The most frequent type of ligament injury is an anterior cruciate ligament (ACL). The mechanisms of an ACL injury are classified as direct contact, indirect contact, and non-contact. Physical examination for the assessment of the ACL is commonly used in routine care in the evaluation of the knee and is part of the diagnostic process. Due to the high degree of variability in their presentation and outcomes, treatment must be tailored according to factors such as patient demographics, the severity of the damage, and long-term improvement profile. When it comes to ACL injuries, low-quality data have been produced that reveals no difference in patient-reported knee function results between surgical ACL restoration and conservative therapy. However, these results must be evaluated in the perspective of the fact that many individuals with an ACL rupture remained symptomatic after rehabilitation and eventually underwent ACL reconstruction surgery. This article has reviewed the risk factors and the mechanisms that commonly lead to ACL injuries. This article has also discussed the clinical significance of conservative and surgical management and has highlighted the implications of both approaches.

## Introduction and background

The anterior cruciate ligament (ACL) is just one of two cruciate ligaments present in the human body and is formed of strong, fibrous material that provides support for its excessive mobility [1]. The purpose of the ACL is to detect the changes in direction of movement, the position of the knee joint, and the changes in speed, acceleration, and rigidity [2]. Most ACL injuries occur along with damage to other structures in the knee, such as articular cartilage, meniscus, or other ligaments [3]. The mechanisms of an ACL injury are classified as direct contact, indirect contact, and non-contact out of which non-contact injuries are the most common and are caused by forces generated within the athlete's body [4]. A cut-and-plant action, which is a quick change in direction or speed with the foot firmly planted, is a common mechanism that causes the ACL to tear [4]. ACL injuries have also been connected to quick deceleration movements, such as planting the afflicted leg to cut and change direction, landing from a jump, pivoting, twisting, and direct impact to the front of the tibia [4]. Many studies have found that female athletes had a greater incidence of ACL injuries than male athletes, owing to differences in physical training, muscular strength, and neuromuscular control [4]. Other possible explanations include pelvic and lower extremity variations, increased ligament looseness, and estrogen's influence on ligament characteristics [4]. Patients with ACL tears complain of hearing or feeling a pop, swelling, significant pain, and joint instability. Physical examination for the assessment of the ACL is commonly used in routine care in the evaluation of the knee and is part of the diagnostic process. The anterior Lachman test (LT), anterior drawer test, and pivot-shift test (PST) are the most well-known physical tests used to assess the ACL's integrity [5]. A physical examination is frequently sufficient to make a diagnosis, although testing may be necessary to rule out other causes and evaluate the severity of the injury. To rule out a bone fracture, X-rays may be necessary. X-rays, on the other hand, do not disclose soft tissues like ligaments and tendons, whereas an MRI reveals the degree of an ACL injury as well as evidence of damage to other tissues in the knee, such as cartilage [6]. The initial goal after an ACL injury is to reduce swelling with ice, elevation, and compression [7-10]. Definitive care often comprises physical therapy or surgical repair to restore mobility and preserve long-term knee function [11]. Since ACL injuries present with a high degree of variability in their presentation, the mode of management must be tailored according to factors such as patient demographics, the severity of the damage, and long-term improvement profile [12]. The focus of this article is to analyze previous medical literature and analyze the best course of treatment for patients suffering an ACL injury based on the above parameters.

## Review

The ACL controls anterior tibial mobility and limits excessive tibial rotation. The ACL is made up of two primary bundles: the posterolateral (PL) and the anteromedial (AM) [2]. Both bundles begin on the posteromedial side of the lateral femoral condyle and terminate immediately anterior to the intercondylar tibial eminence [1]. The AM bundle has an average length of 33 mm, whereas the PL bundle has 18 mm. The moderate ACL cross-sectional area for males and women is 36 and 47 mm^2^, respectively [1-3]. The ACL is constructed of collagen fibers type 1. The primary blood supply to the ligament is provided by the middle genicular artery, with additional supply supplied by the inferomedial and inferolateral genicular arteries [1]. The ACL contains mechanoreceptors such as Ruffini corpuscles, Pacinian corpuscles, Golgi-like structures, and free nerve endings [3].

The most prevalent type of ligament injury in the United States is ACL injury [4]. Although direct contact with the knee can result in injury, the etiology of ACL injury includes causes that do not involve contact. Certain variables put patients at a greater risk including: the feminine sex is linked to an ACL injury as risk factor, due to variations in muscle training, control, and strength, as well as hormonal factors, for that reason the rate of ACL injury in female athletes is three times higher than in male competitors. With the discovery of sex hormone receptors in the ACL, female ligamentous laxity is often greater than male ligamentous laxity, and hormonal activities affect female ligamentous laxity, making female athletes more vulnerable to ACL injury [13]. Males are more likely to sustain contact injuries, whereas females are more likely to sustain non-contact injuries, which may be due to differences in sports participation [14]. Participation in particular sports such as basketball, soccer, football, volleyball, downhill skiing, lacrosse, and tennis, where ACL ruptures are common due to cutting, pivoting maneuvers, and landing on one leg, demands frequent and abrupt deceleration. A previously damaged or improperly repaired ACL [13,14].

It is hypothesized that ACL injuries are caused by both external and internal factors [4]. Movements that disrupt patients’ coordination just prior to landing or deceleration in motion are considered to be the most important external factors [13]. Gender differences in anatomy, higher hamstring flexibility, increased foot pronation, hormonal impacts, and changes in the nerves and muscles that govern knee position are all internal influences [4,5]. Multiplanar movement patterns observed during non-contact injury that are thought to place high strain on the ACL include decreased knee flexion, excessive knee valgus, lateral trunk displacement, and increased hip extension [14]. The risk of re-injuring an ACL that has been repaired is around 15% higher than the chance of tearing a normal ACL [14]. According to one study, the risk is greatest in the first year following the original injury. Also, the risk of an ACL tear in the opposite knee is also higher once the injury has occurred previously; ACL injuries are most frequent between the ages of 15 and 45 years, thus, age is a risk factor as well, owing to a more active lifestyle and increased engagement in sports [13,14].

With a three-month recovery and rehabilitation, the prognosis for a slightly torn ACL is usually favorable. Some patients with partial ACL tears, on the other hand, may still experience symptoms of instability [4,13,14]. Close clinical follow-up and a thorough course of physical therapy help diagnose individuals with unstable knees owing to partial ACL rupture [14].

Nonsurgical

Nonsurgical and surgical treatment plans differ not only in terms of whether patients undergo ACL reconstruction but also in terms of rehabilitation and recommendations for future sports participation. Clinicians are routinely asked to advise patients on whether surgical or nonsurgical treatment is the best option [4,5]. Knowledge of the clinical course following both treatment options is critical for guiding treatment decisions. Individuals who choose conservative treatment must undergo physical therapy to strengthen the muscles around the knee, notably the quadriceps femoris and hamstring muscles [5]. However, in the absence of surgical treatment, the knee remains unstable and vulnerable to injury [5].

In the study conducted by Park et al., 85 patients were selected and studied from day one of injury to a one-year follow-up. Initially, 84% of the patients had a grade 1 LT and a grade 2 PST, whereas 16% had a grade 2. At the one-year follow-up, 77 patients (91%) with LT and PST grade 1 did not require reconstruction, but eight patients with LT or PST grade 2 did (six patients received the operation, and two refused). Patients with LT and PST grade 1 had an average Lysholm score of 91.2, an average side-to-side difference (SSD) of 2.5 mm, and a mean Tegner score of 6.2, down from 6.9 (pre-injury). Patients who began non-operative therapy before two weeks of damage had a higher grade 0 or 1 instability rate than those who started treatment after two weeks (P = 0.043) [15]. This study concluded that it is recommended that non-operative treatment begins within two weeks of an ACL injury with a strict rehabilitation program to build the strength of the injured structures within the knee to achieve optimal results in the remodeling process and healing of the injured site [15]. Comparative research of 48 patients from first therapy to a two-year follow-up by Ahn et al. suggests that non-surgical treatment may assist a subset of individuals with acute ACL damage. There were 12 complete ACL ruptures (25%) and 36 incomplete ACL ruptures (75%). The patients were clinically, and MRI monitored for 21.5 and 11.3 months, respectively. In 41 patients, the follow-up Lachman test improved to grade 0 (87%). In the follow-up pivot shift test, 36 patients (76%) demonstrated no laxity. The most recent follow-up International Knee Documentation Committee (IKDC) score was a mean of 91.1 points. The KT 2000 procedure was carried out on 40 patients, with a mean SSD of 2.85 mm. On MRI, 46 of 48 patients had regained ACL continuity, and 39 (84%) had restored low signal intensity [16]. As a result, it was found that joint laxity on physical examination had improved at the follow-up. These data suggest that non-operative treatment may assist a limited percentage of people with acute ACL damage [16]. In a study of 43 patients from initial treatment to a six-week and then a two-year follow-up, Grindem et al. discovered that surgically treated patients (n = 100) were more likely to participate in level-I sports before injury than nonsurgically treated patients (n = 43). According to the preliminary research, surgically treated patients were more likely to develop a knee re-injury and engage in level-I sports in the second year of the follow-up period. On the other hand, nonsurgically treated individuals were considerably more likely to engage in level-II activities during the first year of the study and level-III sports during the next two years. After two years, 30% of all patients had an extensor strength deficit, 31% had a flexor strength deficit, 20% had patient-reported knee function below the normal range, and 20% had suffered knee re-injury [17]. It was concluded that patients with a nonsurgical approach were significantly more likely to participate in level-II and/or level-III sports during the first year of follow-up. It was also found that some of these patients after two years and a quick return to their perspective sports knee issues began to present signs of knee instability and evaluation for reconstruction was solicited [17]. Kostogiannis et al. piloted a study of 100 individuals having ACL damage over 15 years. Within three years, 40 patients had returned to their pre-injury activity level or greater. According to the Tegner activity scale, the median activity level 15 years after injury had reduced from 7 to 4 (P=0.001). The mean Lysholm knee score at one and three years after the injury was 96 and 95, respectively, but declined to 86 after 15 years (P=0.001). At 15 years, 49 patients had good/excellent outcomes, while 14 had fair (n = 6) or poor function (n = 8). Patients injured in contact sports had a poorer quality-of-life score on the Knee Injury and Osteoarthritis Outcome Score (KOOS) than those injured in noncontact sports (P=0.05). Because of knee problems, 13 of the 67 patients (19%) were re-operated with an arthroscopic surgery [18]. According to the study, due to a major thorough rehab regimen and early damage detection, 67% of the participants in the research did not require ACL repair. At the three-year follow-up, 60% had the same or higher activity level as before the injury, whereas 31% had a lower level of activity. Patients who participated in contact sports at the time of injury had a lower activity level, which had a more significant impact on their subjective quality of life than patients who did not participate in contact sports [18]. In addition, certain patients with an IKDC level-I or -II activity previous to injury may effectively return to the same sporting activities with non-operative care. According to the analysis by Frobell et al. of 121 patients from injury diagnosis to a two-year follow-up, 30 (51%) patients randomized to optional delayed ACL repair experienced delayed ACL reconstruction (seven between two and five years). After adjusting for baseline score, The mean change in KOOS scores from the reference point to five years was 42.9 points for those allocated to therapy plus early ACL repair and 44.9 points for those assigned to rehabilitation alone set to optional delayed reconstruction (between-group difference 2.0 points, 95% confidence interval −8.5 to 4.5; P=0.54 after adjustment for baseline score). At five years, there were no significant changes between groups in the KOOS score (P=0.45), any of the KOOS scale (P=0.12), the SF-36 (P=0.34), the Tegner activity scale (P=0.74), or incidence radiographic osteoarthritis of the index knee (P=0.17) [19]. There were no between-group differences in the number of knees that had meniscus surgery (P=0.48) or in a time-to-event analysis of the fraction of meniscuses operated on (P=0.77). When the data were evaluated by therapy, they were identical [19]. The study determined that ACL reconstruction could be avoided in 61% of patients by providing a well-structured rehabilitation program.

From the different studies mentioned in Table 1, patients who opted into having a conservative treatment, one can conclude that a conservative treatment satisfied most patients when their sporting activities were neither competitive nor could be altered by avoiding contact sports. Rehabilitation after an ACL injury is set to begin during the acute period of the injury. Many physicians’ goals for an ACL injury rehab program include the following: restoring knee range of motion, managing pain, reducing swelling, allowing for early ambulation, and starting muscle strengthening exercises.

**Table 1:** Anterior cruciate ligament conservative treatment ACL: anterior cruciate ligament

References	Design	No of the cases studied	Study Parameters	Diagnostic Criteria	Conclusion
Park et al. [15]	Cohort prognostic study	85	Initial three months treatment with one-year follow-up	MRI clinical evaluation (Lysholm score, Tegner activity score, Lachman test, pivot-shit test).	In the acute period of ACL damage, non-operative therapy using a brace looks to be an effective and practical approach for reaching a satisfactory clinical result. Non-operative treatment should begin within two weeks following an ACL injury to obtain better outcomes in the remodeling process and healing of the damaged location.
Ahn et al. [16]	Cohort prognostic study	48	Initial treatment with a two-year follow-up	MRI clinical evaluation (Lachman test, pivot-shit test, Lysholm score, International Knee Documentation Committee score).	These data show that nonsurgical therapy may assist a subgroup of individuals with acute ACL damage. A considerable improvement was noted when comparing clinal evaluations done during the two-year follow-up to those performed during the first evaluation.
Grindem et al. [17]	Cohort prognostic study	43	Initial treatment for six weeks and a two-year follow-up	MRI clinical evaluation (Isokinetic knee extension and flexion strength, and Sports participation).	This study revealed that patients who used a nonsurgical strategy were considerably more likely to participate in level-II and level-III sports over the first year of follow-up. Knee problems occurred after two years.
Kostogiannis et al. [18]	Cohort prognostic study	100	Initial treatment with a continuous follow-up lasting 15 years	MRI arthroscopy clinical evaluation (Lysholm score, Tegner activity level, and global knee function).	Sixty-seven percent of the participants in the study did not get ACL restoration. At the three-year follow-up, 60% had the same or higher activity level as before the injury, whereas 31% had a lower level of activity.
Frobell et al. [19]	Randomized controlled trial	121	Initial treatment with a two-year follow-up	MRI clinical evaluation (ACL insufficiency, Tegner score 5-9).	In 61% of cases, patients can avoid ACL reconstruction by choosing a well-structured rehabilitation program.

Surgical

Without surgical intervention, complete ACL ruptures have a much poorer prognosis. Several patients cannot participate in cutting or pivoting-type sports after a total ACL tear, while others have instability during even typical tasks such as walking [20]. This diversity is influenced by the degree of the initial knee injury and the patient's physical demands. Approximately half of all ACL injuries are associated with the meniscus, articular cartilage, or other ligament injuries [20]. Secondary damage can occur in patients who have recurrent bouts of instability due to an ACL injury [20]. 

In the study conducted by Laxdal et al., meniscal surgery was performed on 550 (58%) of the 948 patients in the study group before, during, or after ACL restoration. The median Tegner activity level before the injury was 8 (range: 2-10), 3 (range: 0-9) pre-operatively, and 6 (range: 1-10) at follow-up (P=0.0001 pre-operative vs follow-up). The median Lysholm score was 90 points (range: 14-100), the median KT-1000 anterior side-to-side laxity difference was 1.5 mm (range: −6 to 13 mm), and the median one-leg hop test quotient was 95% when compared to the contralateral normal side at follow-up. According to the International Knee Documentation Committee rating method, 69.3% of patients were evaluated as normal or nearly normal at follow-up. However, 36% of the patients were unable to or had significant difficulty executing the knee-walking test. Inferior outcomes were associated with a longer time between the index injury and reconstruction and concurrent joint deterioration discovered during the index procedure [20]. The study demonstrated that a longer time span between initial injury and reconstruction, as well as concomitant joint damage discovered during surgery, was associated with bad outcomes. This was determined after noting 36% of patients were unable or had significant difficulty performing the knee-walking test [20]. The study results of van Dijck et al. showed that 27.6% of the patients underwent a re-intervention. At the time of the procedure, the average age of the 196 patients was 34 years, and the average period of follow-up was 7.4 years. During the 83-month post-surgery period, 77 re-operations were done on 54 (27.6%) patients. Re-interventions were done between day 22 and 83-months post-ACL reconstruction. Indications for re-operations were pain caused by fixation material (n = 25); meniscal lesions (n = 24); cyclops lesion (n = 16); donor site morbidity (n = 5); re-rupture of the ACL (n = 5); posterior cruciate ligament rupture (n = 1); and a medial collateral ligament lesion (n = 1). A more ventral position of the graft on the femur was correlated with a higher frequency of meniscal lesions and cyclops lesions (P<0.01). Patients who had a meniscal lesion after an ACL reconstruction had significantly lower Lysholm (P<0.05) and Tegner scores (P<0.01) [21]. Meniscal lesions, cyclops lesions, donor site morbidity, re-rupture of the ACL, posterior cruciate ligament rupture, and a medial collateral ligament lesion were all reasons for a second intervention. It was determined that surgery at times may produce many complications or instability causing multiple interventions [21]. Pogorzelski et al. discovered that patients who had their grafts resected first had superior postoperative results. Thirty-three (81%) of the 41 patients included in the study were available for follow-up at a mean SD of 54.7±24.4 months and an age of 28.4±9.3 years. Those in group 1 (n = 21) exceeded patients in group 2 (n = 12) on the objective IKDC score (normal or very normal: group 1, 66.6%; group 2, 36.4% ; P=0.047) and KT-1000 measures (group 1, 1.3 1.0 mm; group 2, 2.9 1.5 mm; P=0.005). Group 1 surpassed group 2 on the Lysholm (P =0.007), IKDC subjective (P=0.011), and WOMAC (P=0.069) measures. There was no significant variation in outcomes between groups 2a (n = 4) and 2b (n = 8), despite patients with anterior cruciate ligament graft re-implantation showing a strong propensity toward better results in objective rather than subjective metrics. Magnetic resonance imaging revealed that individuals undergoing graft removal had a greater risk of cartilage injury and meniscal tears than those undergoing graft retention [22]. It was established that graft re-implantation should be performed after ACL reconstruction to avoid future cartilage and meniscal lesions [22]. Drogset et al. study showed that the length of the follow-up was a determining factor to help limit re-injury in patients. Of the remaining 68 patients, the mean Lysholm function score was 84 in the augmentation group and 87 in the control group. There was a statistically significant relationship between pre-operatively detected cartilage injury and osteoarthritis. Almost half of the patients had developed osteoarthritis. We observed no significant difference between the two groups concerning rupture rate, Lysholm or Lachman test scores, or KT-1000 arthrometer measurements [23]. The study's high number of patients who had a second ACL injury, graft rupture, or contralateral ACL rupture may be greater than in the general population. For this reason, a continuous follow-up and good rehab plan can be key factors in diminishing the odds of re-injury [23]. According to the research of Gobbi et al., the fundamental objective of ACL restoration is to return to the same level of sports activity. Sixty-five percent of the 100 patients who underwent ACL repair returned to the same activity level, 24% changed sports, and 11% discontinued sports activities. There was no significant difference in outcome (P>0.05) between PT and HT grafts. Using the IKDC, Lysholm, Noyes, and Tegner knee assessment scales, no significant differences (P>0.05) were found between athletes who "returned" to their former sport and those who "did not return" to sports at the same level. However, there was a difference in knee scores between those who returned to athletics and those who quit entirely. A computerized laxity test found that 90% of these individuals had less than 3 mm of side-to-side variation, with no significant difference between hamstring tendon (HT) and patellar tendon (PT) groups. Patients who returned to sports scored significantly higher. Traditional knee scales such as the IKDC, Lysholm, Noyes, and Tegner continue to be valid for assessing the success after ACL repair [24]. It was reported that 65% of patients returned to the same level of sports performance on average, and only 70% of patients had Tegner activity level drop from initial evaluation to preceding follow-ups [24] (Table 2).

**Table 2:** Anterior cruciate ligament surgical treatment ACL: anterior cruciate ligament, IKDC: International Knee Documentation Committee, WOMAC: Western Ontario and McMaster Universities Osteoarthritis Index, SANE: single assessment numerical evaluation, ACLR: anterior cruciate ligament reconstruction, PT: patellar tendon, HT: Hamstring tendon

References	Design	No. of the cases studied	Study parameters	Diagnostic criteria	Conclusion
Laxdal et al. [20]	Case series	948	Surgery at a median of 12 months (range: 0.5–360 months) after their injury	MRI clinical evaluation (Tegner score, Lysholm score, anterior side-to-side laxity difference, one-leg hop test)	The International Knee Documentation Committee rating method categorized 69.3% of the patients in this research as usual or nearly normal. The knee-walking test, on the other hand, was ineffective or problematic for 36% of patients. A longer time range between the index injury and reconstruction and concurrent joint deterioration revealed was related to worse outcomes during the index procedure.
van Dijck et al. [21]	Case series	196	Surgery with a median follow-up of 32 months	MRI one-incision endoscopic approach with patellar-tendon graft clinical evaluation (detailed history, functional knee ligament testing, KT-1000 arthrometer testing, one-leg-hop testing, Lysholm score, Tegner score, and the IKDC evaluation)	According to the findings of this study, 27.6% of patients required a re-intervention throughout the 83 months following surgery. A second intervention was needed due to meniscal lesions, cyclops lesions, donor site morbidity, re-rupture of the ACL, posterior cruciate ligament rupture, and a medial collateral ligament lesion.
Pogorzelski et al. [22]	Cohort study	41	12 months out from arthroscopic treatment categorized into two groups	MRI clinical evaluation (IKDC evaluation, WOMAC score, Lysholm score)	When compared to patients who underwent initial graft resection, patients with graft retention had better postoperative outcomes. Graft re-implantation should be performed after ACLR to avoid future cartilage and meniscal lesions.
Drogset et al. [23]	Case control	100	Surgery with an average of eight-year follow-up after surgery	MRI clinical evaluation (Lysholm score, Lachman scores, KT-1000 arthrometer measurements) Kennedy ligament augmentation device	A contralateral ACL rupture was recorded in 3–24% of surgically treated individuals, depending on the length of the follow-up. The high proportion of patients in their research who had a future ACL injury, graft rupture, or contralateral ACL rupture may be more significant than in the general population.
Gobbi et al. [24]	Cohort study	100	Surgery (PT or HT) with follow-up at 3, 6, 12, and 24 months	MRI clinical evaluation (IKDC, Lysholm, Noyes, and Tegner score, Marx scale and SANE)	The study showed that 65% of patients returned to the same level of sports performance on average, with only 70% of patients experiencing a drop in Tegner activity level from initial evaluation to subsequent follow-ups.

With further research being conducted to determine whether a surgical approach is the best option, many people believe that pre- and post-operative rehabilitation is critical. These studies suggest that ACL repair does not lower the probability of problems or assure a return to sports. There is no question that ACL rupture can induce knee joint deterioration and re-intervention for a second tear of the ACL joint. Other studies found that patients were able to resume their previous high-level activities, while others found that they had difficulty doing so [25].

Conservative versus surgical

There have been several studies conducted that have compared different patient outcomes when dealing with ACL injuries. Physicians have compared those patients who opted for conservative treatment rather than surgical and those who chose surgical intervention as opposed to conservative treatment.

The study by Meuffels et al. compared the long-term outcomes of highly active patients with ACL ruptures treated surgically versus non-surgically. The conclusion of the study determined that at the time of the examination during follow-up, the patients who had undergone surgical treatment had significantly improved knee stability. However, at a 10-year follow-up, both treatment options show comparable patient outcomes, therefore, no statistical difference between patients treated conservatively or surgically was seen [26]. In a study by Frobell et al., young athletes with an ACL tear were compared between those who received rehabilitation plus early ACL reconstruction and those who received rehabilitation plus ACL reconstruction. This research, which included close consecutive follow-ups, found that rehabilitation plus early ACL replacement did not outperform an initial rehabilitation plan with the possibility of later ACL reconstruction. The outcomes did not differ between those who chose surgical reconstruction early or late versus those who had only received rehabilitation [27]. van Yperen et al. conducted a study to compare the long-term treatment outcomes of operative versus nonoperative ACL rupture treatment in elite athletes [28]. In this retrospective study, it was discovered that after a 20-year follow-up, there was no difference in knee osteoarthritis between operative and a conservative approach when treatment was assigned based on a patient's response to three months of nonoperative treatment [28]. Although the operative group had better knee stability by the next follow-up this was decreased. In the study conducted by Streich et al., 80 patients with arthroscopically proven ACL insufficiency were divided into two groups and followed for 15 years. One half was surgically reconstructed, while the other half was treated with a conservative physiotherapy-based rehabilitation program [29]. Although it is claimed that surgical treatment is superior for restoring overall knee function, the clinical outcomes in this study suggest that outcomes were similar. The assessment scores during clinical evaluations revealed no significant differences between those who had undergone ACL reconstruction versus those who had the physiotherapy-based rehab program [29]. In a trial comprising 121 young, active people with acute ACL damage, Frobell et al. evaluated two strategies: structured therapy plus early ACL restoration versus structured rehabilitation with the option of delayed ACL reconstruction if necessary [19]. A rehabilitation plus early ACL repair method did not outperform a rehabilitation plus optional delayed ACL reconstruction strategy in young, active people with acute ACL injuries [19].

## Conclusions

One of the most often damaged ligaments in the knee is the anterior cruciate ligament. Although ACL injuries manifest with such a wide range of symptoms, the form of treatment must be modified to patient demographics, the severity of the injury, and the patient's long-term recovery profile. Patients with an ACL tear should be advised that surgical repair is not the only option for continuing sporting activities; a conservative approach consisting of a strict and vigorous rehabilitation plan can suffice. The principal purpose of surgery is to enhance knee stability, which can be improved with correct neuromuscular therapy. Whatever therapy they choose, surgical or non-surgical, patients should be advised that the risk of future knee lesions and osteoarthritis remains substantial, especially if they return to high-risk pivoting activities. According to the studies reviewed in this article, surgically treated individuals had a considerably increased chance of re-injuring the knee. Patients in all treatment choices improved significantly in knee function; nevertheless, there were complaints of muscular deficiencies and re-injury at various follow-ups. It is vital to analyze the severity of the injury and advise the patient on the best treatment choice available to obtain a satisfactory outcome. Finally, we urge further research on this issue is conducted to lead to positive patient results.
